# Association of aspartate aminotransferase to alanine aminotransferase ratio with hypertension in Chinese adults: a cross-sectional study

**DOI:** 10.3389/fpubh.2026.1936666

**Published:** 2026-10-22

**Authors:** Xiaoguo Zhang, Hongyan Li, Jingxue Liu, Shengwei Ji, Xue Hu, Zhongfa Zhang

**Affiliations:** 1 Department of Infectious Diseases, Public Health Clinical Center Affiliated to Shandong University, Jinan, China; 2 Public Health Clinical Center Affiliated to Shandong University, Jinan, China

**Keywords:** alanine aminotransferase, aspartate aminotransferase, AST/ALT ratio, Chinese adults, cross-sectional study, hypertension

## Abstract

**Background:**

Emerging evidence has linked the aspartate aminotransferase to alanine aminotransferase (AST/ALT) ratio to insulin resistance and various metabolic disorders, yet its relationship with hypertension in the Chinese adult population has not been well characterized. The present study was designed to evaluate whether the AST/ALT ratio is independently associated with hypertension risk among Chinese adults.

**Methods:**

We conducted a cross-sectional analysis using data obtained from the Dryad repository, originally collected through the Rich Healthcare Group Health screening program. A total of 87,869 participants were enrolled. The AST/ALT ratio was derived by dividing serum AST by ALT concentrations. Hypertension was identified when systolic blood pressure reached ≥140 mmHg or diastolic blood pressure reached ≥90 mmHg. Multivariable logistic regression models with progressive covariate adjustment were employed to assess the AST/ALT ratio–hypertension relationship. Smooth curve fitting coupled with threshold analysis was applied to detect nonlinear patterns, while stratified analyses with multiplicative and additive interaction assessments were performed to examine effect modification. A sensitivity analysis excluding renal-function markers was performed to probe robustness.

**Results:**

A total of 87,869 participants (57.0% male) with a mean age of 42.1 ± 12.8 years were included. In a model adjusted for age, sex, and BMI, each unit increase in the AST/ALT ratio corresponded to a 20% reduction in hypertension odds (OR = 0.80, 95% CI: 0.76–0.84, *p* < 0.001), and the estimate was essentially unchanged in the fully adjusted model (OR = 0.83, 95% CI: 0.78–0.87, *p* < 0.001). When stratified by quartiles, participants in higher AST/ALT ratio categories exhibited progressively lower hypertension risk relative to the lowest quartile (Q2: OR = 0.85, 95% CI: 0.81–0.90; Q3: OR = 0.78, 95% CI: 0.73–0.83; Q4: OR = 0.81, 95% CI: 0.75–0.87). A nonlinear dose–response pattern was identified, with the inflection point occurring at an AST/ALT ratio of approximately 1.010. Stratified analyses demonstrated significant effect modification by age (P for interaction < 0.001) and BMI (P for interaction < 0.001), with stronger inverse associations among individuals younger than 60 years and those with BMI ≥ 25 kg/m^2^.

**Conclusion:**

Among Chinese adults, an elevated AST/ALT ratio is independently and inversely associated with the risk of hypertension, following a nonlinear pattern. Because it is obtainable at no additional cost from routine liver function panels, the AST/ALT ratio may serve as a complementary indicator for flagging individuals who warrant closer blood pressure surveillance; however, the association is modest in size, and the ratio does not replace direct blood pressure measurement.

## Introduction

Hypertension ranks among the most widespread chronic conditions globally and constitutes a principal modifiable determinant of cardiovascular morbidity, cerebrovascular events, renal failure, and premature death (1, 2). Recent global estimates indicate that over 1.28 billion individuals aged 30–79 years are living with hypertension, and this figure continues to rise (3). Within China specifically, survey data reveal that 27.5% of the adult population—approximately 245 million people—are affected, while rates of awareness, treatment, and blood pressure control remain unacceptably low (4). The substantial healthcare burden imposed by hypertension underscores the pressing need for strategies that enable early detection and timely intervention in at-risk individuals.

Insulin resistance (IR) has been recognized as a key pathogenic mechanism linking metabolic disorders and hypertension (5, 6). The hyperinsulinemia that accompanies IR can stimulate sympathetic nervous system activity and activate the renin-angiotensin-aldosterone system (RAAS), enhance renal sodium retention, promote proliferation of vascular smooth muscle cells, and accelerate arterial stiffening—all of which contribute to blood pressure elevation (7, 8). In line with this, surrogate indices of IR have themselves been linked to hypertension in cross-sectional studies; the triglyceride-glucose–waist circumference index, for example, was recently associated with hypertension among Indian adults (9). Nevertheless, the reference standard for IR assessment—the hyperinsulinemic-euglycemic clamp—is costly and operationally demanding, limiting its utility in routine clinical practice. Consequently, there is a clear need for surrogate indicators of IR that are both practical and widely available.

The AST/ALT ratio, originally described by Fernando De Ritis in 1959 (10), has long served as a clinical tool for evaluating hepatic injury and classifying liver diseases. In recent years, accumulating evidence has revealed that the AST/ALT ratio is closely associated with IR and metabolic diseases, including type 2 diabetes mellitus (T2DM), metabolic syndrome, cardiovascular disease, and peripheral arterial disease (11–15). Data from the National Health and Nutrition Examination Survey (NHANES) 2011–2016 demonstrated that a reduced AST/ALT ratio was correlated with heightened IR among US adults (16). In a Chinese cohort, Zhao and colleagues showed that the ALT/AST ratio (the reciprocal of AST/ALT) ranked among the most reliable indicators of IR (17). Additional investigations have established the prognostic relevance of the AST/ALT ratio for incident T2DMe (18–20). Despite these advances, direct evidence linking the AST/ALT ratio to hypertension, particularly in the Chinese population, remains limited currently.

Considering the high prevalence of hypertension in China and the established involvement of IR in blood pressure regulation, we postulated that the AST/ALT ratio, acting as a proxy for IR, might be associated with hypertension risk. Accordingly, the current investigation sought to characterize the association between the AST/ALT ratio and hypertension in a large cohort of Chinese adults using data from the Dryad repository. We wish to be explicit about the intended use case: the AST/ALT ratio is not proposed as a substitute for blood pressure measurement, which remains the diagnostic standard and the basis of existing guideline recommendations. Rather, because liver function panels are routinely obtained in health check-ups and other laboratory encounters, the ratio may serve as a zero-cost, complementary indicator that flags individuals—particularly those whose blood pressure is not measured at a given visit—for closer blood pressure surveillance.

## Methods

### Data sources

Study data were retrieved from the Dryad platform^1^, a repository dedicated to making scientific datasets publicly available, reusable, and citable. The raw dataset was accessed at no cost. In this study, we cited the Dryad data package: Chen Y, Zhang XP, Yuan J, et al. Association of body mass index and age with incident diabetes in Chinese adults: a population-based cohort study (DOI: 10.1136/bmjopen-2018-021768) (21). As the original authors transferred data ownership to Dryad, secondary analysis based on a distinct research question was permissible without infringing upon the authors’ rights. Regarding the original consent context: the source cohort was assembled from the computerized health-screening records of the Rich Healthcare Group; according to the source publication, that study was approved by the Rich Healthcare Group Review Board, the information was retrieved retrospectively, and individual patient consent was not required for the retrospective use of the de-identified records (21). This study followed the principles of the Declaration of Helsinki and the Strengthening the Reporting of Observational Studies in Epidemiology (STROBE) guidelines (22). Given the retrospective nature of this secondary analysis, additional ethical approval was not required.

### Study participants

The source study was a retrospective cohort investigation drawing on health screening records from the Rich Healthcare Group, which operated across 32 sites in 11 Chinese cities (Beijing, Shanghai, Nanjing, Suzhou, Guangzhou, Shenzhen, Changzhou, Chengdu, Wuhan, Hefei, and Nantong). These urban centers span four major geographic regions (Northeast, Southeast, West, and Central China), providing broad population representation. In total, 685,277 adults aged ≥20 years underwent at least two health check-ups between 2010 and 2016. For the present analysis, we performed a cross-sectional evaluation using baseline data. The eligibility criteria were as follows. For their original cohort investigation, the source investigators excluded 473,444 participants who lacked weight or height measurements (*n* = 103,946), lacked gender information (*n* = 1), had extreme BMI values (<15 or >55 kg/m^2^; *n* = 152), lacked fasting plasma glucose values (*n* = 31,370), had visit intervals shorter than 2 years (*n* = 324,233), had diabetes at baseline (*n* = 7,112; 2,997 by self-report and 4,115 by FPG ≥ 7.0 mmol/L), or had undefined diabetes status at follow-up (*n* = 6,630), leaving 211,833 participants. These criteria—the two-visit requirement, the minimum 2-year interval, and the exclusion of participants with diabetes at baseline—were design features of the source investigation of incident diabetes; they are preconditions of the shared dataset rather than choices made for the present analysis. For the present analysis, complete measurements of AST, ALT, SBP, and DBP were required; we sequentially excluded participants with missing AST (*n* = 123,290), missing ALT (*n* = 660), missing SBP (*n* = 12), or missing DBP (*n* = 1), as well as one participant with an implausible AST value of 0 U/L, yielding a final analytic sample of 87,869 (Figure 1). Missing values for covariates were processed using multiple imputations with 5 replications. The exposure (AST/ALT ratio) and the outcome (SBP/DBP) required complete measurement and were handled by the listwise exclusions detailed above; among the covariates, age, sex, BMI, and FPG had no missing values, and the imputed covariates, with their missingness in the analytic sample of 87,869, were: TC and TG (*n* = 1,103, 1.3% each), HDL-C (*n* = 38,175, 43.5%), LDL-C (*n* = 37,336, 42.5%), BUN (*n* = 7,310, 8.3%), Scr (*n* = 3,520, 4.0%), and smoking and drinking status (*n* = 65,847, 74.9% each). Because attendance was employment-based, the source population consisted of employees undergoing occupational health screening rather than a community-based random sample of adults.

**Figure 1:**
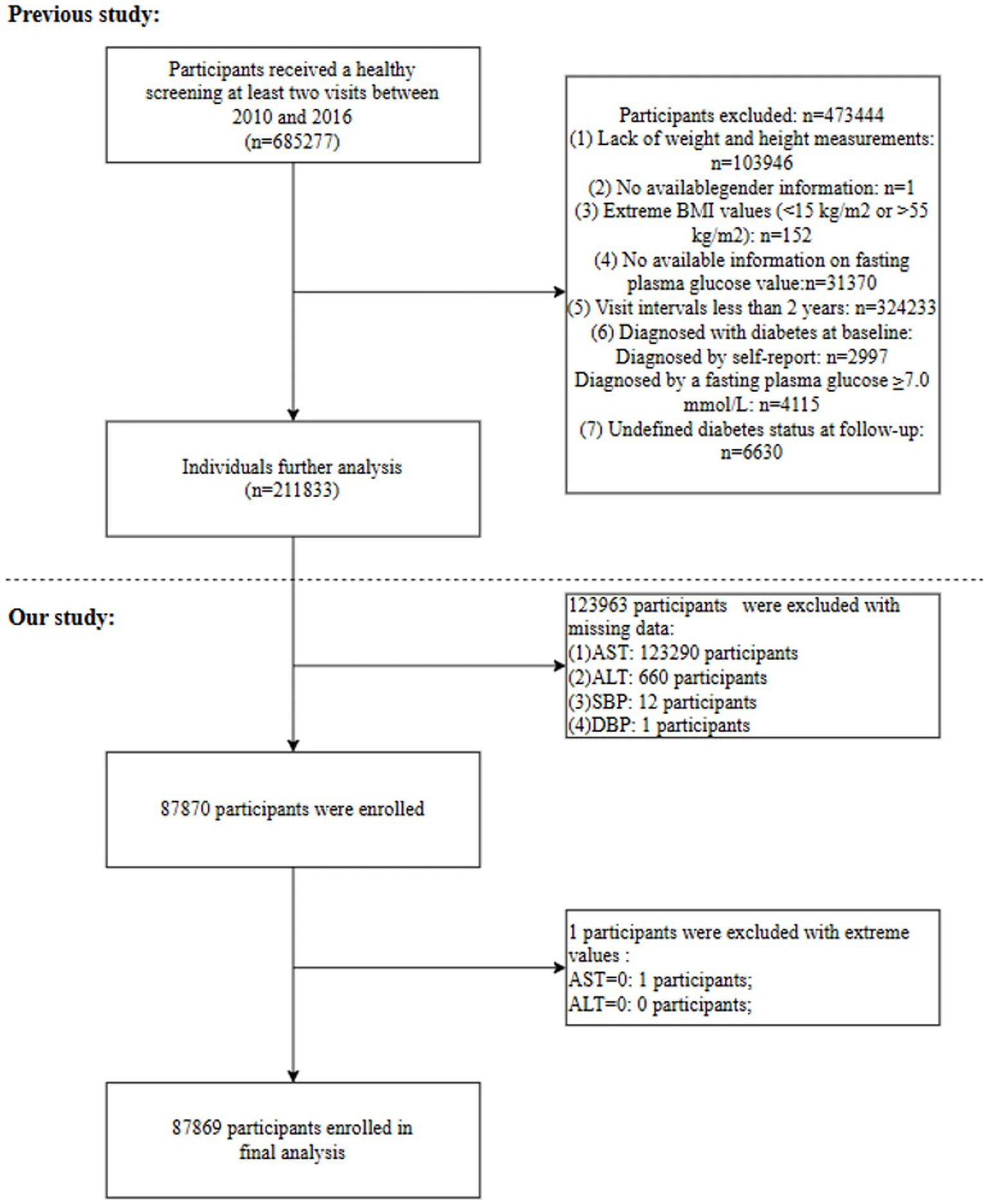
Flow chart of the study population.

### Measurements

Participants completed structured questionnaires capturing demographic characteristics (age, sex), lifestyle factors (smoking and alcohol consumption), medical and medication history, and family history of chronic conditions. Anthropometric indices (height, weight) and blood pressure were recorded by trained personnel, with BMI computed as weight (kg) divided by height squared (m^2^). Blood pressure was obtained using a standard mercury sphygmomanometer. The source documentation does not specify whether blood pressure was recorded as a single reading or as the average of repeated readings, nor the cuff sizes or rest periods applied (21). Following a minimum 10-h overnight fast, venous blood specimens were drawn and analyzed for serum triglycerides (TG), total cholesterol (TC), high-density lipoprotein cholesterol (HDL-C), low-density lipoprotein cholesterol (LDL-C), AST, and ALT on a Beckman 5,800 autoanalyzer. Fasting plasma glucose (FPG) was quantified via the glucose oxidase method on the same platform. The source dataset did not record intercurrent acute illness, recent alcohol intake, strenuous exercise before phlebotomy, recent muscle injury, or recent weight change; such transient factors can alter aminotransferase activities independently of chronic hepatic or metabolic status.

The AST/ALT ratio was computed as the serum AST concentration divided by the serum ALT concentration. Hypertension was defined as SBP ≥ 140 mmHg and/or DBP ≥ 90 mmHg.

### Statistical analysis

Continuous variables with a normal distribution were summarized as mean ± standard deviation (SD), whereas those with a skewed distribution were reported as median (interquartile range, IQR). Categorical variables were expressed as counts and percentages. Group comparisons employed one-way ANOVA or the Kruskal–Wallis H test for continuous data and the chi-square test for categorical data.

Univariate logistic regression was initially performed to screen for variables associated with hypertension. Multivariable logistic regression was then applied in a sequential modeling strategy: the crude model did not adjust for any confounders. Model 1 adjusted for age, gender, and BMI. Model 2 additionally adjusted for FPG, TC, TG, HDL-C, LDL-C, BUN, and Scr. Model 3 further adjusted for smoking status and drinking status. Because FPG and the lipid fractions may themselves lie downstream of the insulin resistance that the AST/ALT ratio is thought to reflect, their causal role in this analysis—confounders versus mediators—is ambiguous; Model 1, which adjusts only for age, sex, and BMI (none of which plausibly lies on this pathway), is therefore presented as a co-primary estimate alongside the fully adjusted model. Sensitivity analysis treating the AST/ALT ratio as a categorical variable (quartiles) was conducted to confirm the robustness of findings. Because BUN and serum creatinine may partly lie on the causal pathway linking long-standing hypertension to the exposure–outcome association, a further sensitivity analysis refitting Model 3 without these two renal-function covariates was performed to gauge the potential impact of over adjustment.

Smooth curve fitting was employed to visualize the dose–response pattern between the AST/ALT ratio and hypertension. A two-piecewise linear regression model was fitted to detect threshold effects, with the inflection point identified through a recursive algorithm and validated by the likelihood ratio test. Stratified analyses were performed across age (<60 vs. ≥60 years), sex (male vs. female), BMI (<25 vs. ≥25 kg/m^2^), smoking status, and drinking status. Both multiplicative and additive interactions were evaluated. The additive interaction was assessed using three indicators: relative excess risk due to interaction (RERI), attributable proportion due to interaction (AP), and synergy index (SI).

All statistical analyses were performed using R statistical software^2^ and Free Statistics software version 2.4 A two-sided *p*-value <0.05 was considered statistically significant.

## Results

### Baseline characteristics of the study population

A total of 87,869 participants (57.0% male) with a mean age of 42.1 ± 12.8 years were included in the study (Table 1). The mean BMI was 23.3 ± 3.3 kg/m^2^, and the mean AST/ALT ratio was 1.2 ± 0.5. When participants were categorized into quartiles based on the AST/ALT ratio, all baseline characteristics differed significantly across groups (*p* < 0.05). Those in the highest quartile (Q4) displayed the lowest values for BMI, SBP, DBP, TG, LDL-C, ALT, BUN, and Scr. Moreover, the proportions of male participants, current smokers, and current drinkers declined progressively across ascending AST/ALT ratio quartiles.

**Table 1:** Baseline characteristics of participants according to quartiles of AST/ALT ratio.

Characteristics	Total	AST/ALT ratio quartile				*p* value
		Q 1 (<0.9)	Q 2 (0.9–1.2)	Q 3 (1.2–1.6)	Q 4 (>1.6)	
Participants	87,869	21,967	21,966	21,965	21,971	
Age (years)	42.1 ± 12.8	40.2 ± 10.3	43.1 ± 12.5	43.1 ± 13.5	42.1 ± 14.3	<0.001
Gender, *n* (%)						<0.001
Male	50,087 (57.0)	18,778 (85.5)	14,548 (66.2)	10,165 (46.3)	6,596 (30.0)	
Female	37,782 (43.0)	3,189 (14.5)	7,418 (33.8)	11,800 (53.7)	15,375 (70.0)	
BMI (kg/m^2^)	23.3 ± 3.3	25.4 ± 3.2	23.7 ± 3.1	22.5 ± 2.9	21.5 ± 2.7	<0.001
SBP (mm Hg)	119.3 ± 16.5	123.4 ± 15.4	120.5 ± 16.3	117.9 ± 16.6	115.5 ± 16.8	<0.001
DBP (mm Hg)	74.2 ± 10.9	77.3 ± 10.8	75.0 ± 10.7	73.0 ± 10.6	71.4 ± 10.4	<0.001
FPG (mmol/L)	4.9 ± 0.6	5.0 ± 0.6	5.0 ± 0.6	4.9 ± 0.6	4.9 ± 0.6	<0.001
TC (mmol/L)	4.7 ± 0.9	4.9 ± 0.9	4.7 ± 0.9	4.6 ± 0.9	4.6 ± 0.9	<0.001
TG (mmol/L)	1.1 (0.7, 1.6)	1.5 (1.0, 2.2)	1.1 (0.8, 1.7)	0.9 (0.7, 1.4)	0.8 (0.6, 1.2)	<0.001
HDL-C (mmol/L)	1.4 ± 0.3	1.3 ± 0.2	1.3 ± 0.3	1.4 ± 0.3	1.4 ± 0.3	<0.001
LDL-C (mmol/L)	2.7 ± 0.7	2.8 ± 0.7	2.7 ± 0.6	2.7 ± 0.6	2.6 ± 0.6	<0.001
ALT (U/L)	24.1 ± 22.6	45.9 ± 34.7	22.7 ± 8.3	16.1 ± 6.6	11.6 ± 5.0	<0.001
AST (U/L)	24.1 ± 12.4	29.7 ± 17.5	23.3 ± 7.9	21.6 ± 8.3	21.7 ± 11.5	<0.001
BUN (mmol/L)	4.7 ± 1.2	4.9 ± 1.1	4.8 ± 1.2	4.6 ± 1.2	4.5 ± 1.2	<0.001
Scr (μmol/L)	71.2 ± 15.9	76.7 ± 13.8	73.1 ± 15.9	68.9 ± 15.5	66.1 ± 16.0	<0.001
AST/ALT ratio	1.2 ± 0.5	0.7 ± 0.1	1.0 ± 0.1	1.3 ± 0.1	1.9 ± 0.5	<0.001
Smoking status, *n* (%)						<0.001
Current smoker	13,411 (15.3)	5,507 (25.1)	3,903 (17.8)	2,494 (11.4)	1,507 (6.9)	
Ever smoker	1,679 (1.9)	724 (3.3)	489 (2.2)	286 (1.3)	180 (0.8)	
Never smoker	72,779 (82.8)	15,736 (71.6)	17,574 (80.0)	19,185 (87.3)	20,284 (92.3)	
Drinking status, *n* (%)						<0.001
Current drinker	958 (1.1)	275 (1.3)	259 (1.2)	212 (1.0)	212 (1.0)	
Ever drinker	8,727 (9.9)	3,607 (16.4)	2,417 (11.0)	1,628 (7.4)	1,075 (4.9)	
Never drinker	78,184 (89.0)	18,085 (82.3)	19,290 (87.8)	20,125 (91.6)	20,684 (94.1)	

Values are mean ± SD, median (IQR), or number (percentage). Missing covariate values were multiply imputed before summarization; age, sex, BMI, and fasting plasma glucose were fully observed. AST, aspartate aminotransferase; ALT, alanine aminotransferase; BMI, body mass index; SBP: systolic blood pressure; DBP: diastolic blood pressure; FPG, fasting plasma glucose; TC, total cholesterol; TG, triglyceride; HDL-C, high-density lipid cholesterol; LDL-C, low-density lipid cholesterol; BUN, blood urea nitrogen; Scr, serum creatinine.

### Comparison of included and excluded participants

Because 123,964 of the 211,833 eligible participants (58.5%) were excluded from the analytic sample—predominantly for missing aminotransferase values—we compared the included and excluded groups on the baseline characteristics measured in both groups (Table 2). Age (42.1 ± 12.8 vs. 42.1 ± 12.6 years; *p* = 0.24), HDL-C (*p* = 0.06), and LDL-C (*p* = 0.57) did not differ significantly between the groups, and FPG, TC, and TG were identical at the precision reported. The remaining differences were nominally significant given the size of the comparison but small in absolute terms: the excluded group contained 3.7 percentage points fewer men (53.3% vs. 57.0%) and had marginally lower BMI (23.2 ± 3.4 vs. 23.3 ± 3.3 kg/m^2^), BUN (4.6 ± 1.2 vs. 4.7 ± 1.2 mmol/L), and Scr (69.2 ± 15.6 vs. 71.3 ± 16.0 μmol/L). The exclusions bore almost entirely on the exposure rather than the outcome: 123,290 of the 123,964 excluded participants (99.5%) lacked an AST measurement, 660 lacked an ALT measurement, and only 13 were excluded for missing blood pressure (12 SBP, 1 DBP).

**Table 2:** Comparison of key baseline characteristics between included and excluded participants.

Characteristic	Included (*n* = 87,869)	Excluded (*n* = 123,964)	*P* value
Age (years)	42.1 ± 12.8	42.1 ± 12.6	0.24
Male, n (%)	50,087 (57.0)	66,036 (53.3)	<0.001
BMI (kg/m^2^)	23.3 ± 3.3	23.2 ± 3.4	<0.001
FPG (mmol/L)	4.9 ± 0.6	4.9 ± 0.6	<0.001
TC (mmol/L)	4.7 ± 0.9	4.7 ± 0.9	<0.001
TG (mmol/L)	1.1 (0.7, 1.6)	1.1 (0.7, 1.6)	<0.001
HDL-C (mmol/L)	1.4 ± 0.3	1.4 ± 0.3	0.06
LDL-C (mmol/L)	2.8 ± 0.7	2.8 ± 0.7	0.57
BUN (mmol/L)	4.7 ± 1.2	4.6 ± 1.2	<0.001
Scr (μmol/L)	71.3 ± 16.0	69.2 ± 15.6	<0.001

Values are mean ± SD, median (IQR), or number (percentage). *p* values are from *t* tests (continuous variables) or the chi-square test (categorical variables).

### Univariate logistic regression analysis

Univariate analyses (Table 3) identified age, BMI, FPG, TC, TG, LDL-C, ALT, AST, BUN, Scr, and the AST/ALT ratio as significant correlates of hypertension (all *p* < 0.05). Female sex, elevated HDL-C, never smoking, and never drinking were each associated with reduced hypertension odds. Notably, the AST/ALT ratio exhibited an inverse relationship with hypertension (OR = 0.56, 95% CI: 0.54–0.59, *p* < 0.001).

**Table 3:** Results of univariate logistic regression analysis.

Variable	Odds ratio (95% CI)	*p* value
Age (years)	1.06 (1.06 ~ 1.06)	<0.001
Gender		
Male	Reference	
Female	0.51 (0.49 ~ 0.53)	<0.001
BMI (kg/m^2^)	1.22 (1.22 ~ 1.23)	<0.001
FPG (mmol/L)	2.15 (2.08 ~ 2.22)	<0.001
TC (mmol/L)	1.43 (1.40 ~ 1.46)	<0.001
TG (mmol/L)	1.43 (1.41 ~ 1.45)	<0.001
HDL-C (mmol/L)	0.62 (0.58 ~ 0.67)	<0.001
LDL-C (mmol/L)	1.53 (1.49 ~ 1.57)	<0.001
ALT (U/L)	1.01 (1.01 ~ 1.01)	<0.001
AST (U/L)	1.02 (1.02 ~ 1.02)	<0.001
BUN (mmol/L)	1.23 (1.21 ~ 1.25)	<0.001
Scr (μmol/L)	1.02 (1.02 ~ 1.02)	<0.001
AST/ALT ratio	0.56 (0.54 ~ 0.59)	<0.001
Smoking status		
Current smoker	Reference	—
Ever smoker	0.79 (0.69 ~ 0.91)	0.001
Never smoker	0.66 (0.63 ~ 0.69)	<0.001
Drinking status		
Current drinker	Reference	—
Ever drinker	0.42 (0.36 ~ 0.49)	<0.001
Never drinker	0.34 (0.30 ~ 0.39)	<0.001

BMI, body mass index; FPG, fasting plasma glucose; TC, total cholesterol; TG, triglyceride; HDL-C, high-density lipid cholesterol; LDL-C, low-density lipid cholesterol; ALT, alanine aminotransferase; AST, aspartate aminotransferase; BUN, blood urea nitrogen; Scr, serum creatinine.

### Association between the AST/ALT ratio and hypertension in multiple regression models

Multivariable logistic regression results are presented in Table 4. Without covariate adjustment, the AST/ALT ratio showed a marked crude inverse association with hypertension (OR = 0.56, 95% CI: 0.54–0.59, *p* < 0.001); however, the ratio was strongly patterned by many factors (Table 1), so this unadjusted estimate was heavily confounded, and our conclusions rest on the adjusted estimates below. After controlling for age, sex, and BMI (Model 1), the inverse association persisted (OR = 0.80, 95% CI: 0.76–0.84, *p* < 0.001). Further adjustment for metabolic biomarkers in Model 2 slightly attenuated the effect (OR = 0.84, 95% CI: 0.80–0.89, *p* < 0.001). In the fully adjusted Model 3, which additionally accounted for smoking and drinking, the AST/ALT ratio remained inversely associated with hypertension (OR = 0.83, 95% CI: 0.78–0.87, *p* < 0.001). The association was thus directionally identical and of similar magnitude with or without the metabolic covariates (Model 1 vs. Model 3: OR = 0.80 vs. 0.83), so the conclusion does not depend on whether FPG and the lipid fractions are treated as confounders or as mediators. In the sensitivity analysis excluding BUN and serum creatinine, the association was essentially unchanged (OR = 0.82, 95% CI: 0.78–0.87, *p* < 0.001), indicating that the inverse association is not an artifact of adjustment for renal function.

**Table 4:** Association between AST/ALT ratio and hypertension in multiple regression model.

Exposure	Crude Model		Model 1		Model 2		Model 3	
	OR (95% CI)	*P*	OR (95% CI)	*P*	OR (95% CI)	*P*	OR (95% CI)	*P*
AST/ALT ratio	0.56 (0.54–0.59)	<0.001	0.80 (0.76–0.84)	<0.001	0.84 (0.80–0.89)	<0.001	0.83(0.78–0.87)	<0.001
AST/ALT ratio quartiles								
Q1	Reference	—	Reference	—	Reference	—	Reference	—
Q2	0.78 (0.74–0.82)	<0.001	0.83 (0.79–0.88)	<0.001	0.86 (0.82–0.91)	<0.001	0.85(0.81–0.90)	<0.001
Q3	0.59 (0.56–0.62)	<0.001	0.76 (0.71–0.80)	<0.001	0.80 (0.75–0.85)	<0.001	0.78(0.73–0.83)	<0.001
Q4	0.49 (0.47–0.52)	<0.001	0.77 (0.72–0.83)	<0.001	0.82 (0.77–0.88)	<0.001	0.81(0.75–0.87)	<0.001
P for trend	0.79 (0.77 ~ 0.8)	<0.001	0.91 (0.89 ~ 0.93)	<0.001	0.93 (0.91 ~ 0.95)	<0.001	0.93(0.90 ~ 0.95)	<0.001

Results are shown as odds ratios (ORs) with 95% confidence intervals (CIs). AST, aspartate aminotransferase; ALT, alanine aminotransferase. Crude model: adjusted for none. Model 1: Adjusted for Age, Gender, BMI. Model 2: Adjusted for the variables in Model 1 plus FPG, Cholesterol, TG, HDL-C, LDL-C, BUN, Scr. Model 3: Adjusted for the variables in Model 2 plus Smoking status, Drinking status.

When the AST/ALT ratio was analyzed as a categorical variable (quartiles), a similar trend was observed. Compared with the lowest quartile (Q1), the risk of hypertension decreased from Q2 to Q4 in the fully adjusted model (Q2: OR = 0.85, 95% CI: 0.81–0.90; Q3: OR = 0.78, 95% CI: 0.73–0.83; Q4: OR = 0.81, 95% CI: 0.75–0.87). The P for trend was significant across all models (*p* < 0.001).

### Nonlinear relationship between the AST/ALT ratio and hypertension

Smooth curve fitting revealed a nonlinear dose–response pattern between the AST/ALT ratio and hypertension after full covariate adjustment (P for nonlinearity < 0.001) (Figure 2). The risk of hypertension decreased with increasing AST/ALT ratio, but the slope of the curve attenuated at higher AST/ALT values. Threshold analysis (Table 5) further delineated this nonlinear pattern: when the AST/ALT ratio was less than 1.010, each unit increase in the AST/ALT ratio was associated with a 26.2% reduction in the risk of hypertension (OR = 0.738, 95% CI: 0.603–0.904, *p* = 0.003), whereas above this threshold, the inverse association was markedly weaker (OR = 0.929, 95% CI: 0.864–0.998, *p* = 0.044). The likelihood ratio test confirmed the existence of a nonlinear relationship (*p* < 0.001).

**Figure 2:**
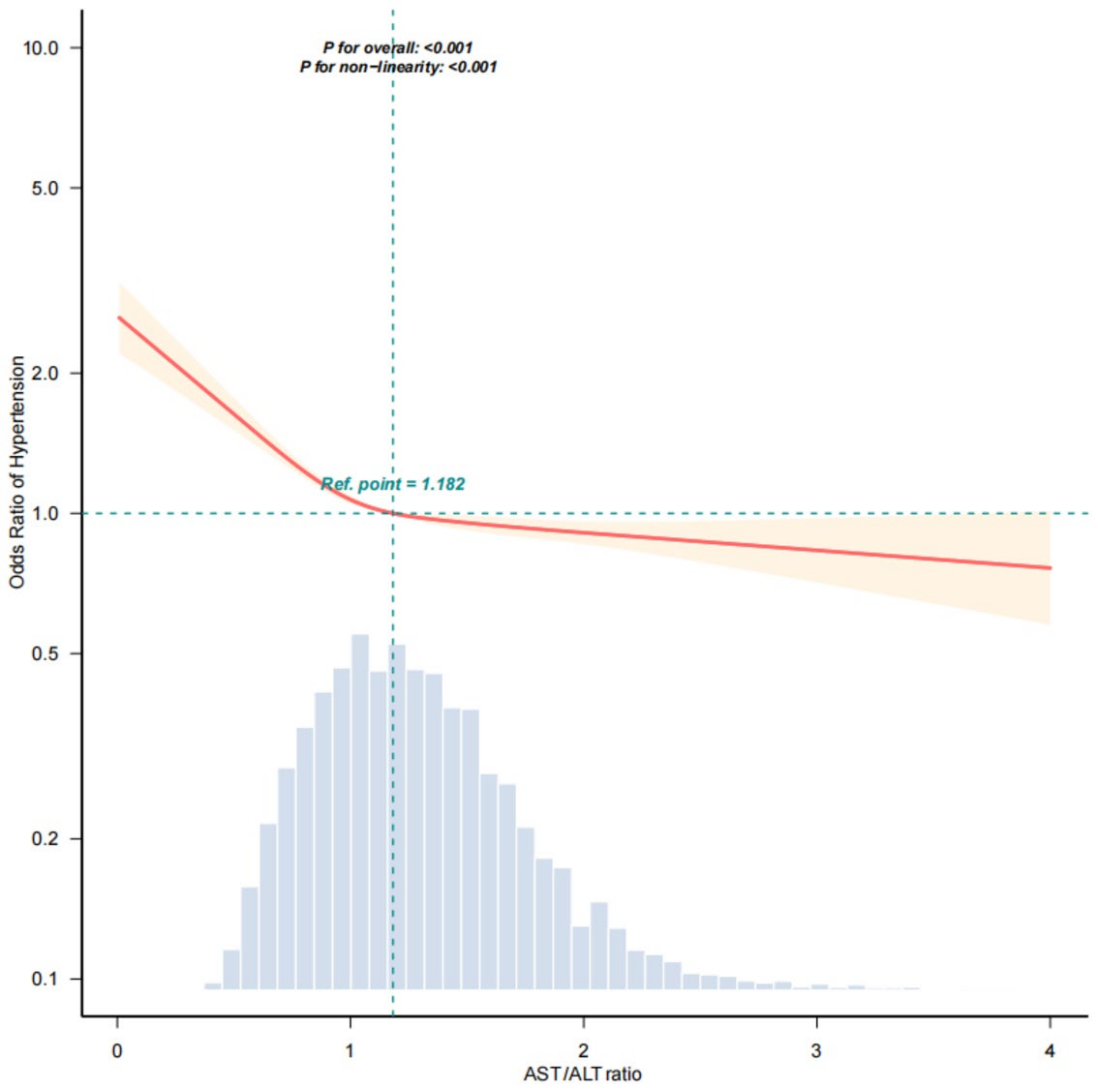
A nonlinear relationship of AST/ALT ratio and hypertension. the model was adjusted for Age, Gender, BMI, FPG, Cholesterol, TG, HDL-C, LDL-C, BUN, Scr, Smoking status, Drinking status.

**Table 5:** Inflection point analysis of AST/ALT ratio and hypertension.

Item	Breakpoint/OR (95%CI)	*P* value
Estimate. breakpoint.1	1.010(1.001,1.018)	
Effect_size_1	0.738 (0.603 ~ 0.904)	0.0033
Effect_size_2	0.929 (0.864 ~ 0.998)	0.0435
Likelihood ratio test	—	<0.001
Non-linear test×1	—	0.001
Non-linear test×2	—	<0.001

Adjusted for Age, Gender, BMI, FPG, Cholesterol, TG, HDL-C, LDL-C, BUN, Scr, Smoking status, Drinking status.

### Subgroup analyses and interaction tests

Stratified analyses were performed to further investigate the influence of other risk factors on the association between the AST/ALT ratio and hypertension (Figure 3, Table 6). The negative association between the AST/ALT ratio and hypertension was consistent across all subgroup variables. However, significant interactions were observed for age and BMI.

**Figure 3:**
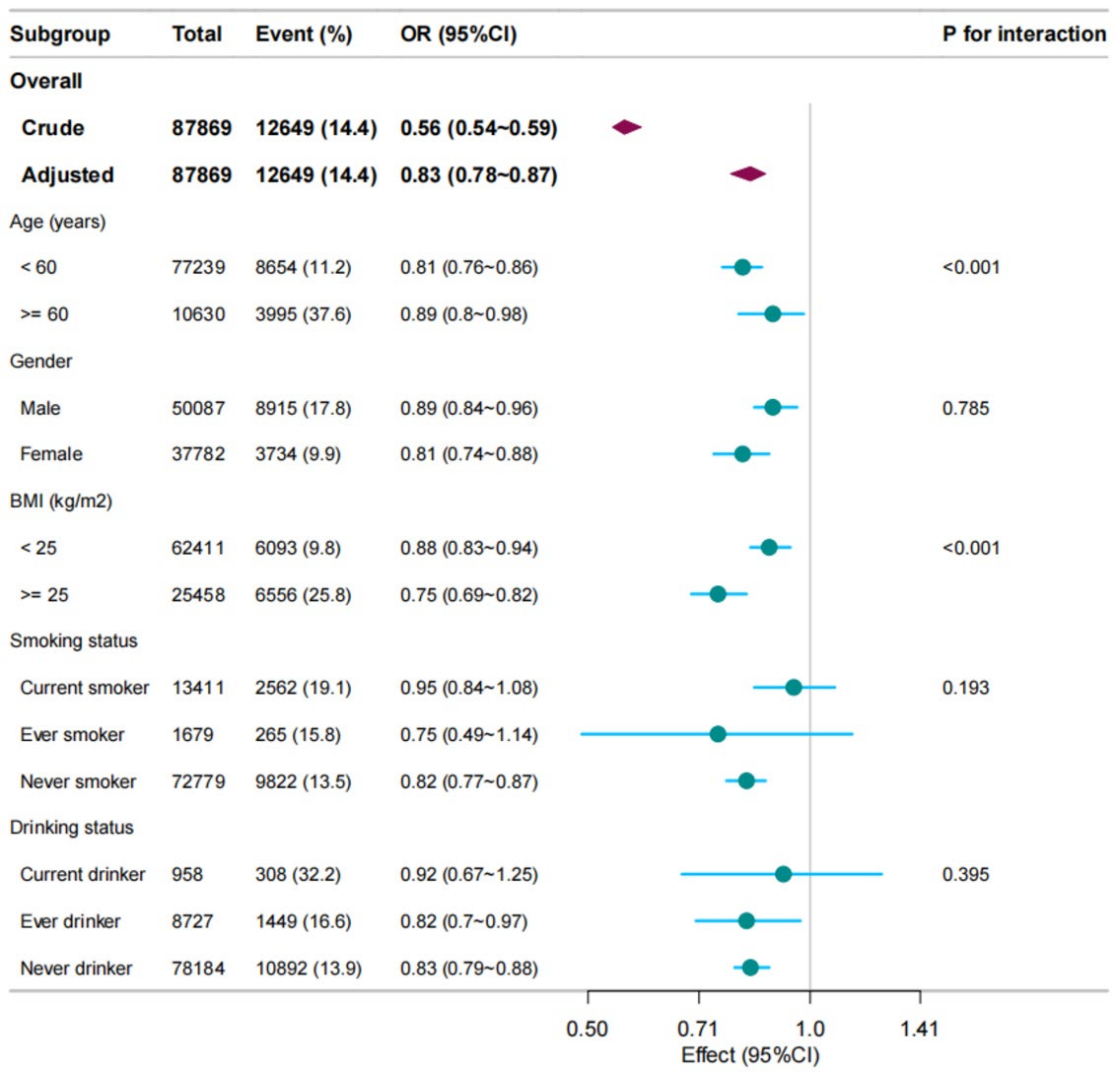
Association between AST/ALT ratio and hypertension in different subgroups. Except for the stratification component itself, each stratification factor was adjusted for Age, Gender, BMI, FPG, Cholesterol, TG, HDL-C, LDL-C, BUN, Scr, Smoking status, Drinking status.

**Table 6:** Multiplicative and additive interactions between age, BMI and AST/ALT ratio on hypertension.

Item		AST/ALT ratio	Multiplicative interaction		Additive interaction		
			OR (95% CI)	P	RERI (95% CI)	AP (95% CI)	SI (95% CI)
Age^*^ (years)			1.57 (1.42 ~ 1.73)	<0.001	1.12 (0.81 ~ 1.42)	0.27 (0.21 ~ 0.33)	1.55(1.37 ~ 1.76)
	<60	low					
	≥60	high					
BMI^**^ (kg/m^2^)			0.91 (0.83 ~ 1)	0.05	−0.36 (−0.51 ~ −0.22)	−0.22(−0.32 ~ −0.12)	0.64(0.53 ~ 0.77)
	<25	low					
	≥25	high					

*The analysis was adjusted for Gender, BMI, FPG, Cholesterol, TG, HDL-C, LDL-C, BUN, Scr, smoking status, drinking status. **The analysis was adjusted for Age, Gender, FPG, Cholesterol, TG, HDL-C, LDL-C, BUN, Scr, smoking status, drinking status.

For the age subgroup, the negative association was stronger in participants aged <60 years (OR = 0.81, 95% CI: 0.76–0.86) than in those aged ≥60 years (OR = 0.89, 95% CI: 0.80–0.98), with a significant multiplicative interaction (OR = 1.57, 95% CI: 1.42–1.73, *p* < 0.001) and additive interaction (RERI = 1.12, 95% CI: 0.81–1.42; AP = 0.27, 95% CI: 0.21–0.33; SI = 1.55, 95% CI: 1.37–1.76).

For the BMI subgroup, the negative association was stronger in participants with BMI ≥ 25 kg/m^2^ (OR = 0.75, 95% CI: 0.69–0.82) compared with those with BMI < 25 kg/m^2^ (OR = 0.88, 95% CI: 0.83–0.94), with a significant multiplicative interaction (OR = 0.91, 95% CI: 0.83–1.00, *p* = 0.05) and additive interaction (RERI = −0.36, 95% CI: −0.51 to −0.22; AP = −0.22, 95% CI: −0.32 to −0.12; SI = 0.64, 95% CI: 0.53–0.77). For sex, the inverse association was present in both males (OR = 0.89, 95% CI: 0.84–0.96) and females (OR = 0.81, 95% CI: 0.74–0.88) in the fully adjusted model, with no significant multiplicative interaction (*p* = 0.785); no significant interactions were detected for smoking status (*p* = 0.193) or drinking status (*p* = 0.395).

## Discussion

In this large-scale cross-sectional investigation encompassing 87,869 Chinese adults, we observed an independent inverse association between the AST/ALT ratio and hypertension prevalence after comprehensive covariate adjustment. This relationship was robust across different modeling strategies and remained evident whether the AST/ALT ratio was modeled as a continuous or categorical variable. Moreover, we uncovered a nonlinear dose–response pattern characterized by an inflection point at an AST/ALT ratio of approximately 1.010, indicating that the inverse association of a higher AST/ALT ratio is most evident at lower ratio values and wanes beyond this threshold. Stratified analyses identified age and BMI as significant effect modifiers, with younger participants (<60 years) and those with elevated BMI (≥25 kg/m^2^) exhibiting a more pronounced inverse association. A further point concerns the composition of the quartiles: the exposure and sex were strongly correlated (85.5% of the lowest AST/ALT quartile were male vs. 30.0% of the highest), so the sex-adjusted estimates leaned heavily on within-sex contrasts. The consistent within-sex associations argue that the overall finding is not an artifact of this compositional skew, although some imprecision in the higher quartiles, where males were the minority, cannot be excluded.

These findings align with prior research linking the AST/ALT ratio to cardiometabolic outcomes. Wang and colleagues reported that an elevated AST/ALT ratio was associated with reduced diabetes risk in Chinese adults with prediabetes (18). In a Japanese cohort, Nakajima et al. identified a low AST/ALT ratio as a predictor of incident diabetes (19). Liu et al. documented an inverse relationship between the AST/ALT ratio and peripheral arterial disease among Chinese hypertensive patients (20). Additionally, Liu et al. demonstrated that the AST/ALT ratio was inversely associated with all-cause and cardiovascular mortality in hypertensive individuals (23). Collectively, these observations support the concept that the AST/ALT ratio may constitute a useful indicator for identifying individuals at elevated cardiometabolic risk. However, direct investigations of the AST/ALT ratio in relation to hypertension have been scarce, and the present study extends the literature by establishing a negative and nonlinear association in a large Chinese adult population. The nonlinear nature of this relationship is particularly noteworthy, as it indicates that the inverse association is more pronounced when the ratio is below the inflection point of 1.010 and diminishes beyond this threshold. This observation delineates the range within which the ratio carries the most information, although whether a change in the ratio corresponds to a change in hypertension risk can be established only prospectively.

Several biological pathways may underpin the observed inverse association. The AST/ALT ratio has been validated as a surrogate indicator of IR, with a lower ratio reflecting greater hepatic fat accumulation and more extensive liver injury—both of which are closely tied to IR (16, 17). In this cohort, the quartile gradient was driven predominantly by ALT (45.9 ± 34.7 U/L in the lowest quartile vs. 11.6 ± 5.0 U/L in the highest, a roughly four-fold range) while AST varied comparatively little (29.7 ± 17.5 vs. 21.7 ± 11.5 U/L)—a pattern consistent with hepatic steatosis, in which the predominantly hepatic-cytosolic ALT rises while AST changes little. Conversely, an AST-driven elevation of the ratio—typically reflecting alcohol intake or muscle injury, given the extrahepatic expression of AST in skeletal muscle, myocardium, and erythrocytes—is unlikely to account for the observed gradient in this cohort, where current drinkers constituted only 1.1% of participants. A low ratio in this population therefore primarily marks hepatic steatosis and the associated insulin-resistant phenotype. Once present, IR can raise blood pressure through at least three interconnected mechanisms: first, compensatory hyperinsulinemia activates the sympathetic nervous system and RAAS, leading to increased sympathetic tone and vasoconstriction (7, 8); second, hyperinsulinemia enhances renal sodium reabsorption, resulting in volume expansion and elevated blood pressure (24, 25); and third, IR promotes vascular smooth muscle cell proliferation and collagen deposition, contributing to arterial stiffness and increased peripheral vascular resistance (26, 27).

This study has several methodological strengths. The large sample drawn from multiple urban centers across China provides substantial statistical power and enhances the generalizability of the findings within the Chinese adult population. The sequential multivariable modeling and sensitivity analyses reduce the likelihood that the observed associations are driven by confounding. The application of smooth curve fitting and inflection point analysis offers a more nuanced understanding of the dose–response relationship. Furthermore, comprehensive stratified analyses with both multiplicative and additive interaction assessments reveal important population subgroups in whom the AST/ALT ratio may be most informative. From a practical standpoint, the AST/ALT ratio is readily derived from routine liver function tests; however, the fully adjusted association is modest in size (OR = 0.83 per unit), and the multiplicative interaction with BMI borders on significance (*p* = 0.05). We therefore make no claim of clinical screening value for the ratio. At most, it may serve as a complementary risk-flagging indicator within panels that are ordered anyway, and it cannot substitute for direct blood pressure measurement. This study also has several limitations. First, the cross-sectional design precludes causal inference; longitudinal cohort studies are needed to determine whether the AST/ALT ratio predicts incident hypertension. Reverse causation also cannot be excluded: hepatic steatosis can lower the AST/ALT ratio and promote hypertension through shared insulin-resistant pathways, so a low ratio may accompany, rather than precede, elevated blood pressure. Second, some important confounding variables, such as physical activity, dietary factors, and antihypertensive medication use, were not available in the database and could not be adjusted for. Comorbid illness was likewise unavailable for adjustment, although comorbid conditions are common in hypertensive populations and may relate to both aminotransferase levels and blood pressure (28). Third, the AST/ALT ratio was measured only at baseline, precluding assessment of longitudinal trajectories. Fourth, as a secondary analysis, we relied on measurement procedures carried out by the original data collectors across 32 sites in 11 cities between 2010 and 2016; the source publication reports standardized data collection by trained staff and laboratory methods with rigorous internal and external quality control (21), but we cannot independently verify inter-site analyzer calibration or uniform adherence to a single blood pressure protocol, including the number of readings per visit, cuff sizes, and rest periods. Fifth, AST is not liver-specific, and intercurrent illness, recent alcohol intake, strenuous exercise before phlebotomy, muscle injury, and recent weight change can transiently alter the AST/ALT ratio; because these acute factors were not recorded in the source dataset, they may introduce noise or misclassification into the exposure measurement. Sixth, the source population comprised employees attending occupational health screening, who are likely to differ from the general adult population in income, education, occupation type, and healthcare access; such factors can independently influence BMI, diet, and blood pressure, and the healthy-worker effect may also apply. Seventh, the study population was restricted to Chinese adults, and the findings may not be directly transferable to other ethnic or racial groups: reference values of the AST/ALT ratio vary with ethnicity, body composition, alcohol consumption patterns, and the background prevalence of hepatitis B and C, and the inflection point of 1.010 identified here should be regarded as cohort-specific until it is externally validated in other populations. Eighth, the analytic sample was reached through layered exclusions: of the 211,833 eligible participants, 123,964 were excluded, predominantly for missing aminotransferase values (123,290 lacked AST, 660 lacked ALT). The included and excluded groups were closely matched on the characteristics measured in both (Table 2), but residual selection bias cannot be excluded; and because the inherited source-cohort criteria restrict the sample to diabetes-free, health-screening-attending adults, the present estimates may not transfer to populations that include individuals with diabetes. Relatedly, missing covariate values were handled by multiple imputation, which assumes missingness at random conditional on observed data; because the proportion of missing smoking and drinking data was high (74.9% each) and imputed values are model-based, sensitivity to imputation-model assumptions cannot be ruled out.

## Conclusion

In summary, this study provides evidence that the AST/ALT ratio is independently and inversely associated with the risk of hypertension in Chinese adults, with a nonlinear dose–response pattern that plateaus beyond an AST/ALT ratio of 1.010. The modifying effects of age and BMI indicate that the inverse association is most evident among younger and overweight individuals. Given that it is already available at no additional cost in routine laboratory panels, the AST/ALT ratio may serve as a complementary indicator that flags individuals warranting closer blood pressure surveillance; the association is modest, however, and the ratio is not a screening instrument and cannot substitute for direct blood pressure measurement. Further prospective studies are warranted to confirm these findings and to clarify the mechanistic pathways involved.

## Data Availability

The datasets presented in this study can be found in online repositories. The names of the repository/repositories and accession number(s) can be found at: 10.1136/bmjopen-2018-021768.

## Footnotes

1
www.datadryad.org

2
http://www.R-project.org
